# Notes from the Conference of Educational Associations

**Published:** 1922-02

**Authors:** E. M. Field


### NOTES FROM THE CONFERENCE OF EDUCATIONAL ASSOCIATIONS.

By Mrs. E. M. FIELD.

FIFTY-TWO separate associations joined in this year's conference in London, and as the Women Teachers were meeting elsewhere and a Northern conference was also in progress, it was evident that teachers and educationists were busily studying their work. From such discussions certain points naturally stand out and mark the trend of thought, and this year the fact that the National League for Health took "Adolescence" as its general subject did not stand alone. Speakers at other meetings took up points which showed the new underlying conviction that childhood and youth are not merely preparations for later life, but have a completeness of their own. The schoolchild is not merely a human tadpole, a future citizen, but a being to whom stress and responsibility, and the impulse of enjoyment and action, have already come. The period of adolescence is, in fact, one for which, as one speaker wisely said, older people should feel more sympathy than they do, remembering their own experiences of difficulty and instability. Growth, in fact, involves hard work in extracting nourishment from food and air, and using it to build up the body, and this work has to be done in adolescence concurrently with that of mental training and the acquisition of physical control and skill. The young, in fact, need more protection that we are giving them, and this in a variety of ways. For instance, Mr. R. E. Roper put in an extremely interesting plea, founded on his own experience at Eton, and at Bedales, for more opportunities of rest and even of sleep for growing boys. A set of height and weight charts showed in six cases studied through the adolescent years how irregular is the progress of growth, at times so rapid as to exhaust strength and energy, and then stationary or even retrograde, an examination sometimes causing actual lowering of the height, an unhappy holiday a loss of weight. Some shadow drawings showed bulges of the spine at times of overstrain. Mr. Roper pleaded strongly that games and ever more games are not the one panacea for all ills of youth. Others have declared them not sufficiently constructive to satisfy some minds. He would have a medical examination before any athletic training, especially into the condition of the heart, would replace long races by short ones, and forbid swimming under water, and any attempts to win the *silver* life-saving medal before the age of 18 or 19; above all, he would let a boy lie down and sleep when he feels the need of doing so. The tendency to overwork in an eager boy or girl should be watched and restrained.

---

Dr. Woodhouse Gloyne touched the same note from another standpoint when he spoke of the "diseases and disabilities" of the adolescent. More links are needed, in his view, between the school doctor and the parents. Protection here is against the lighting-up of inherited diseases or sequelæ of childish illnesses. Eye-trouble may arise when the more intense study of books begins, deafness may prove a severe handicap. Children, said more than one of the speakers on feeble-mindedness, are sometimes supposed "dull" because they do not hear half of what is said by their teachers. Dr. Auden, of Birmingham, raised a very difficult question when he spoke of the handicap of epilepsy and that of stammering, each a serious bar to progress and success in life. Epileptics whose fits are infrequent are, he considers, greatly in need of discipline and the regularity of school life, and should not be excluded, while the stammerer should only be kept away for a few days at a time if his trouble becomes so severe as to make rest necessary. He refuses to admit that other children may be adversely affected by the presence either of the stammerer or the epileptic, and it would be interesting both to teachers and social workers to be officially told whether or not these troubles are "catching."

---

Another question of protection was raised by Dr. Saleeby, namely, that such protection is due to young people during a stage of life marked by emotional instability and the emergence of an instinct for joyous life, excitement and gaiety which is not blameworthy. The father in Charpentier's opera "Louise," who thought it sufficient recreation for a young daughter to read the newspaper aloud, forgot the impulses of his own youth. Societies, Dr. Saleeby thinks, work vainly for the suppression of diseases while the young are thrown unguarded and often unwarned into streets that bristle with temptations. In New York the Association for Social Hygiene has accomplished a solid and evident improvement by providing the positive influences for good of pleasant social intercourse with mixed dancing, games and varied occupations of many kinds, with the addition of a stern repression of flaunted prostitution and the diminution, if not abolition, of facilities for getting drink, that champion weakener of self-control. According to Stanley Hall, the three motives usually appealed to for avoidance of wrong-doing are fear, the prospect of future parenthood and the instinct of self-protection. But a number of speakers at various meetings set the matter on a much higher plane of thought. It is claimed that in New York the miserable cases so sadly common there of amateur prostitutes of 14 or less, promptly affected by disease and often for long concealing it, are now practically unknown. For us, effort in this matter is extremely important. Last year the repeated plea was for organised sex instruction. This year its strongest advocate, Miss March, urged that care should be exercised lest it should be given too fully at too early an age, and sex should seem to fill the whole landscape to the child itself, as to certain psycho-analysts.

**Notes from the Conference of Educational Associations—(*cont.*).**

Dr. Winifred Cullis pointed out the beauty and interest which reside in the processes of growth. Those which she instanced were mostly too advanced for educational use, as the experiments in parthenogenesis which have produced the real frog, the feeding and disease-filtering work of the placenta, and the barrier raised by the fertilised ovum against further entry of spermatozoa. But there is little doubt that early lessons in rudimentary natural science, especially when made living by keeping of pets and visits to a Zoo, are the best avenue to a pure joy in life and its manifestations, and presently its reproduction. Miss Maude Royden put forcibly the gross injustice of allowing young people to suffer, as study and inquiry have shown that many do, from a horrified sense of abnormality and something like personal disgrace over perfectly natural symptoms. Furtive hints and grimy ways of satisfying what is a perfectly proper curiosity are things which in truth defile the mind, while young minds respond readily to the idea of home and parenthood hereafter.

---

How early such ideas stir in the mind a true anecdote may show. Said a child of three lately to her mother, after putting her doll to sleep, " I'm going to buy a baby."

" Very nice, but how will you take care of it ? You are only a baby yourself." A pause.

" When I am eleven I shall buy a baby."

" That's better. But you'll have to buy a daddy first to work for it." A longer pause.

Then : " How much did you give for your man when you boughted him ? "

---

Dr. Pembrey threw something of a bomb into his audience by asserting stoutly that after 14 co-education is a snare and delusion. Girls need then to be taught by women, and to grow into womanly women, preferred so by men. Boys need men at that time ; in games the association of girls keeps them back, while it may overstrain the girl. His studies deduce much from the opposite sex impulses which can be induced by surgical operation. The gentleness and plumpness of the eunuch, the cockerel that was seen to " mother " a brood of chickens, and the occasional deplorable development of the unsexed woman are significant. Mr. Green's discourse on the psychical history of adolescence accorded with this in its reference to a stage of quarrelling with the opposite sex which comes before the time of love affairs, and sometimes develops those " raves " for a teacher or friend of the same sex, which, at times, make the adolescent something of a ridiculous and tiresome person.

---

But the possible sublimation of this feeling by the development of the " sheepdog " instinct was delightfully brought out several times, notably so in the lecture of Mr. Ernest Young on care by elder " brothers " and " sisters " of the younger. Wonderful things are being done by *Toc. H.* in its three houses in London and others in provincial towns, where young ex-soldiers go to stay, being bound by the promise to do social work of some kind, in a boys' club, or where else needed. And two masters of elementary schools gave delightful accounts of the school camps and journeys, in which they and others, with the help of volunteers, all young, forge this invaluable link. Such, again, is the work of the men and boys of the Sheffield Rovers, who began by taking a few little boys for change to their own camp on breezy hills. Is not here the truest and best kind of remedy for depravity and disease ?

---

The question of sufficient nourishing food was raised by Professor Mottram, and gives, indeed, food for thought. Scientific study has now gone far to show the factors, and " accessory food factors," as it is now the fashion to call vitamines, which may be found in cheap or dear food, in cabbage at 4d. or asparagus at 26s. Prof. Mottram has valued out a suggested dietary on the cheapest lines which he proposes as foundation at least for a minimum wage. It is a useful idea, and greatly interested the teachers of Domestic Science, before whom the lecture was given.

---

### HOSPITAL COMMITTEES IN SCOTLAND.

TWO Local Voluntary Hospitals Committees have so far been set up in Scotland, one for Glasgow and the other for the Highlands and Islands area. The latter is working under peculiar conditions, for there already exists a State Fund from which the voluntary hospitals in the Highlands and Islands of Scotland may receive assistance. The Highlands and Islands Medical Service Fund was established by the Treasury in 1913, with the object of assisting in the provision of an adequate medical service for that area, which is three-fourths the total area of Scotland. It is now administered by the Scottish Board of Health, which, in co-operation with the local authorities, general practitioners and voluntary agencies, is operating several schemes (*e.g.*, general practitioner scheme, nursing scheme, ambulance scheme, &c.), having in view a complete medical service. The whole arrangements are striking as an example of successful co-operation of the public authorities with private agencies. Several of the voluntary hospitals have already received grants, and the new Voluntary Hospitals Committee cannot ignore the existence of this Fund in their consideration of claims from the Highlands hospitals. It appears to us that, especially in view of the existence of the Highlands and Islands (Medical Service) Fund, the main problem of the Highlands hospitals is not finance but transport. The distribution of the Highlands population is scattered, and more or less isolated communities make the question of transport especially important. The difficulties arising therefrom, we are assured, can be solved by co-operation ; and the principal task of the new Committee under the chairmanship of the Duchess of Atholl will be to stimulate this co-operation.

---

According to the " Correspondencia España," a bill has been prepared by the Minister of the Interior for introduction into the Spanish Parliament by which persons intending to marry will be compelled to produce a medical certificate showing that both are in good health.

